# An infective endocarditis complicated by multiple septic emboli: case report

**DOI:** 10.1186/s43044-024-00451-z

**Published:** 2024-02-10

**Authors:** Meriam Amri, El Mehdi Tamir, Abdenasser Drighil, Rachida Habbal

**Affiliations:** Cardiology Department, CHU Ibn Rochd, Casablanca, Morocco

**Keywords:** Infective endocarditis, Septic emboli, Brain abscess, Splenic infarction, Spinal emboli

## Abstract

**Background:**

Owing to challenges associated with heart failure and cardiac embolism, infectious endocarditis emerges as a critical pathology characterized by elevated mortality and morbidity rates. Our case stands out as a rare instance of endocarditis involving multisystem embolization, with a successful outcome.

**Case presentation:**

We present the case of an 81-year-old man whose admission was further complicated by various septic emboli affecting the brain (manifesting as a brain abscess and ischemic stroke), spleen (resulting in splenic infarction), and spinal cord. The patient received a diagnosis of infective endocarditis affecting the native mitral valve. Following prompt medical and surgical intervention, the overall progression was favorable despite encountering several challenges.

**Conclusions:**

This case is notable for its detailed description and analysis of the multiple embolic events. More importantly, it underscores the significance of timely surgical intervention and the collaborative approach of a heart team in the face of complicated endocarditis marked by numerous septic emboli. Despite the typically grim prognosis associated with such cases, the outcomes emphasize the positive impact of timely surgery on prognosis.

**Supplementary Information:**

The online version contains supplementary material available at 10.1186/s43044-024-00451-z.

## Background

Despite being rare (about 30 cases per million), infectious endocarditis (IE) is exceedingly dangerous, with a 15–25% hospital mortality rate or at three months [1]. The left heart’s valves are more severely impacted, and embolic events make the prognosis worse. Surgery is recommended in the acute phase after hemodynamic, septic, or thromboembolic issues when medical care is well administered. In our case, the patient’s improvement despite the poor prognosis is what makes it interesting.

## Case presentation

**Fig. 1:**
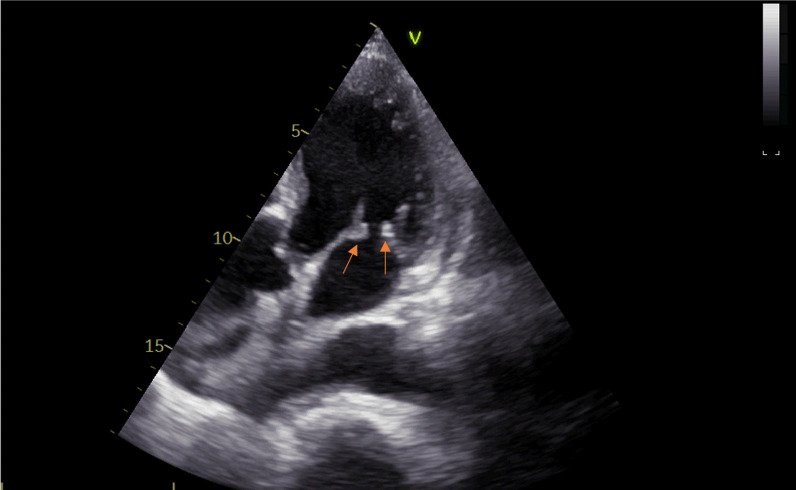
Echocardiographic image showing the 2 vegetations on the 2 mitral valves

**Fig. 2:**
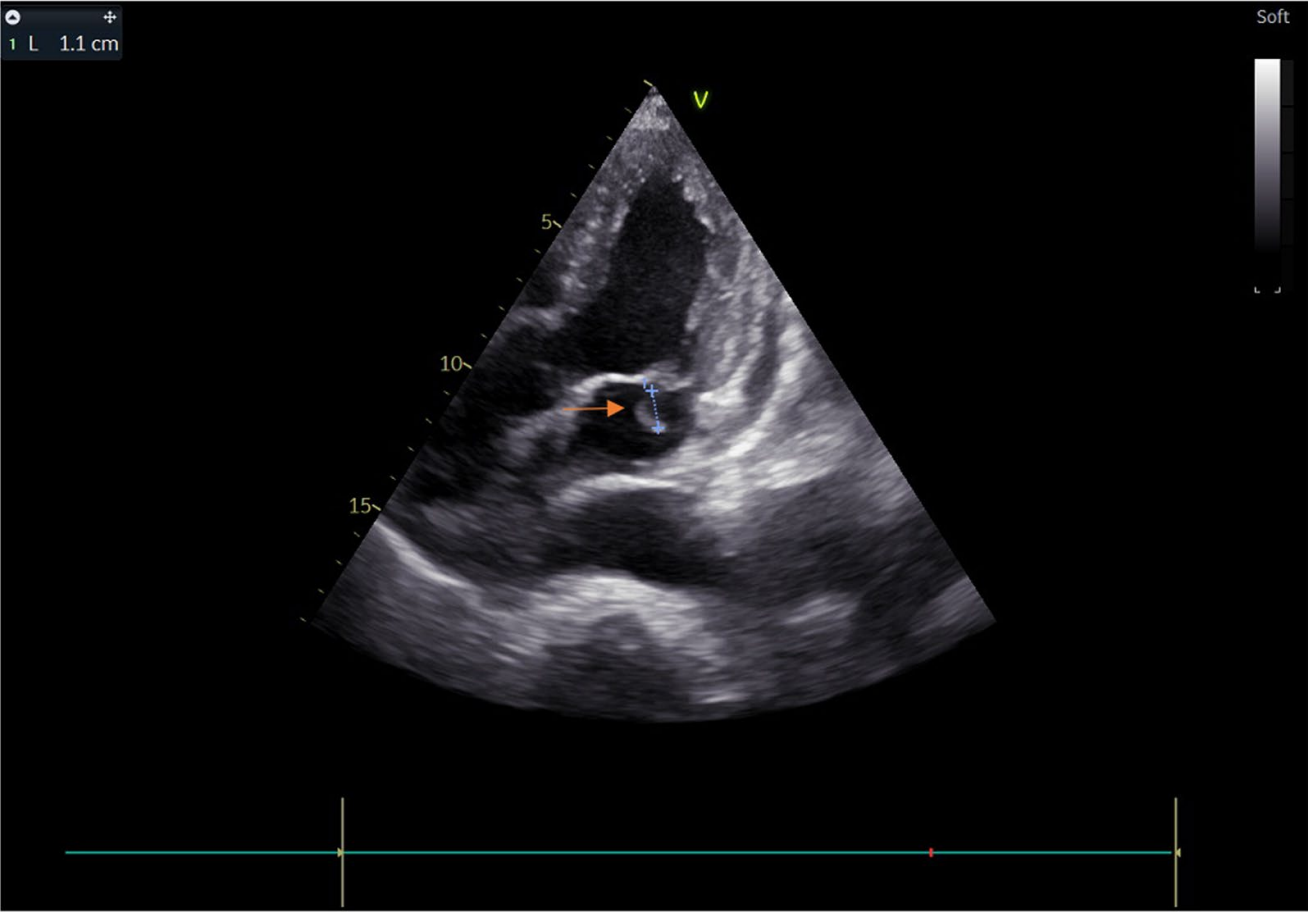
Echocardiographic image showing mitral vegetation on the atrial side

**Fig. 3:**
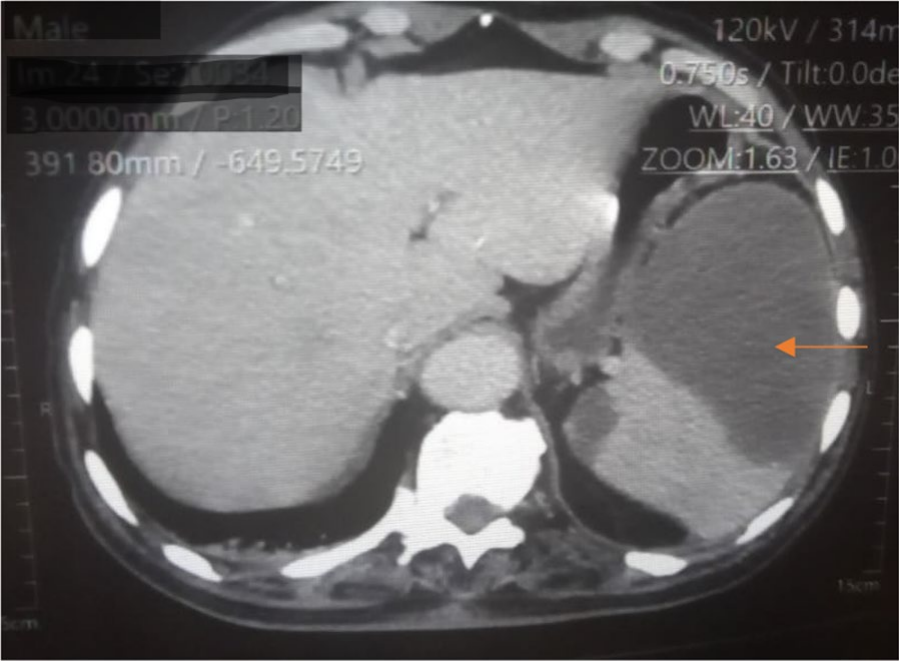
Abdominal scan showing splenic infarction

**Fig. 4:**
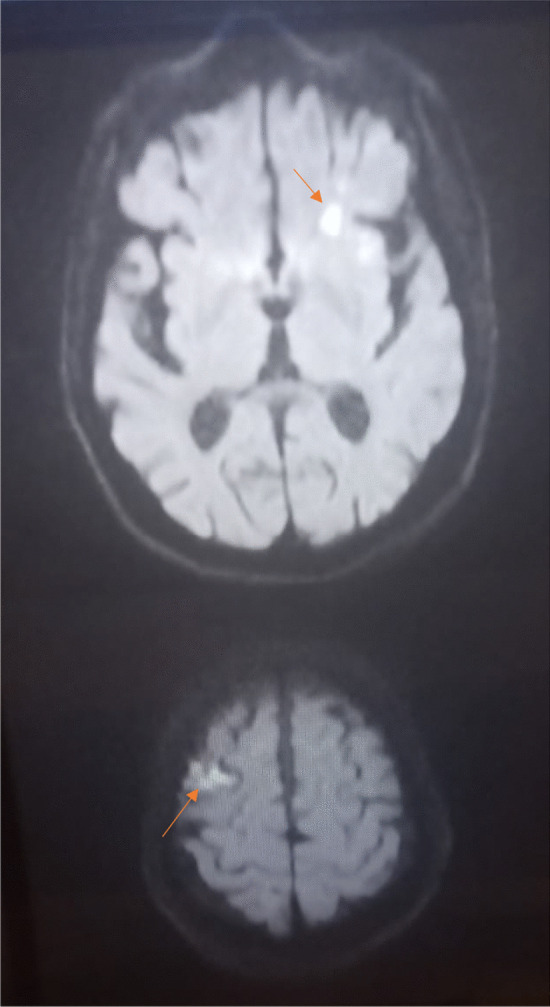
Brain MRI showing a right frontal lesion evoking a cerebral abscess and a left parietal lesion evoking an ischemic stroke at the subacute stage

**Fig. 5:**
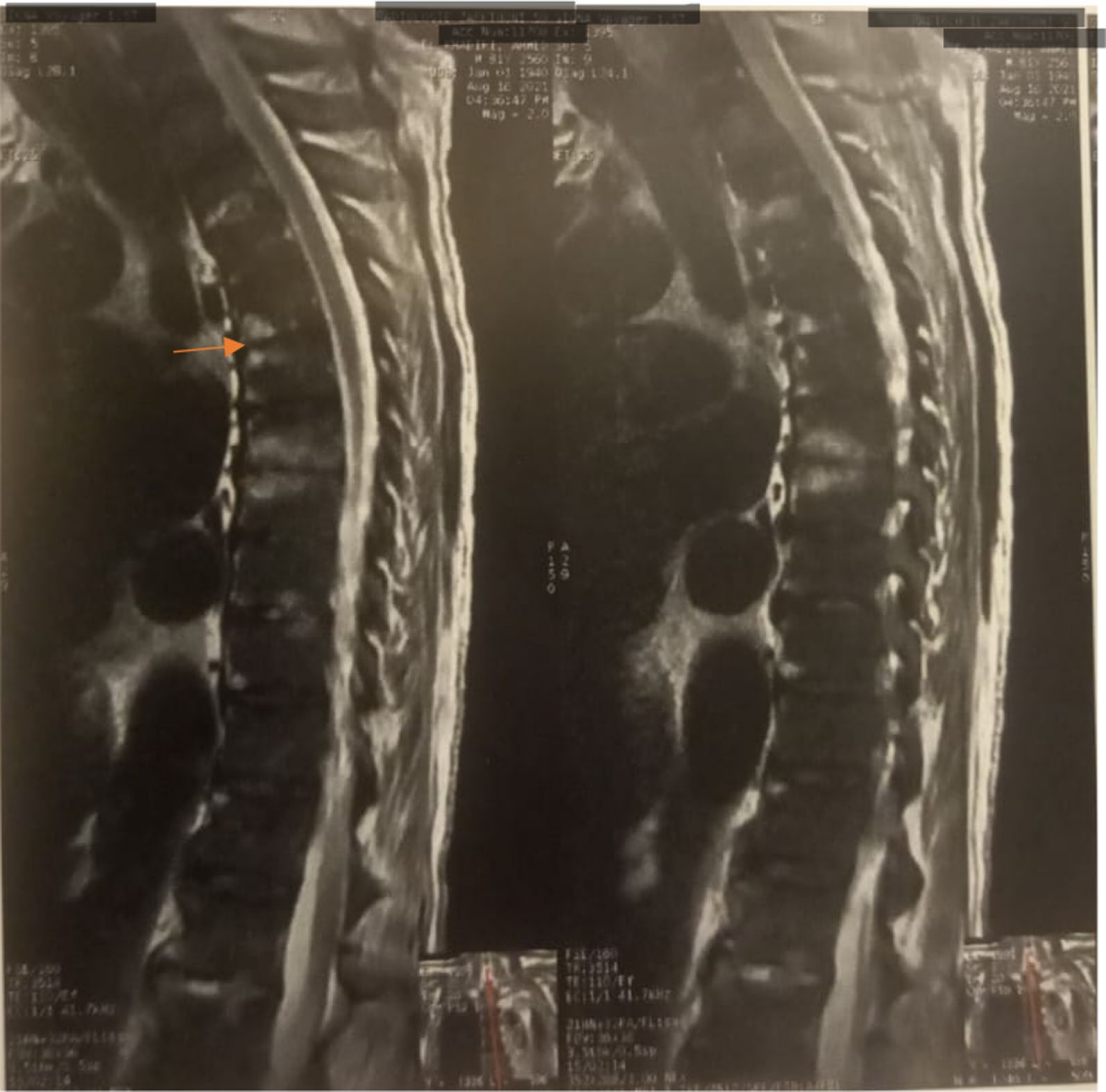
Cervico-dorso-lumbar MRI showing vertebral osteomyelitis

An 81-year-old diabetic man with asthenia and no known cardiac disease was admitted for a chronic fever persisting for over a month. Additionally, he reported pain in his left hypochondrium and presented with an altered general condition. Upon the physical examination, the patient was found to be awake, febrile (38.3 °C), and hemodynamically stable. According to the New York Heart Association, he had dyspnea stage II. During the cardiovascular assessment, a 3/6th systolic murmur at the mitral focus was detected. Neurological testing revealed left hemiparesis. Abdominal examination was normal, with no evidence of hepatosplenomegaly. Blood work showed a hemoglobin level of 135 g/L, a white blood cell count of 14.45 × 109/L, with a predominance of neutrophils (10.92 × 109/L), and a platelet count of 252 × 109/L. The C-reactive protein level was elevated at 116.3 mg/L. Serum electrolytes, creatinine levels, liver enzyme levels, and the coagulation profile were all within normal ranges. On echocardiography, two vegetations were discovered, one measuring 11*10 mm on the anterior mitral leaflet and another measuring 14*12 mm on the posterior mitral leaflet. These vegetations had thickened the mitral valve seating, resulting in severe mitral regurgitation (Jet area/Left atrium area ratio = 45%, vena contracta = 9 mm, EROA by PISA = 40 mm^2^) (Figs. 1 and 2) (In video, severe mitral regurgitation and vegetations are provided in additional files 1 and 2). Three sets of blood cultures were conducted as follows: three pairs of bottles (one aerobic and one anaerobic) for each set, with each bottle containing 10 ml of blood drawn from a peripheral vein under strict aseptic conditions. These sets were spaced at least 1 h apart and taken at the time of the thermal peak. Two distinct sets of blood cultures grew Aerococcus Viridans, which was susceptible to: penicillin (MIC = 0.009 mg/l), vancomycin (MIC = 0.25 mg/l), and ceftriaxone (MIC = 0.5mg/l). Additionally, no source of infection or portal of entry was identified. A thoraco-abdomino-pelvic CT scan revealed hypodense splenic lesions with thick fluid density associated with an older infarction (Fig. 3). Brain MRI showed bilateral ischemic lesions, with a right frontal lesion suggesting a cerebral abscess and a left parietal lesion consistent with a subacute ischemic stroke (Fig. 4). Cervico-dorso-lumbar MRI revealed lesions on the two sacral fins and the iliac wings, predominantly on the left, without spinal cord discomfort, as well as hyperintense signals in the dorsal cervical vertebral bodies, indicative of vertebral osteomyelitis (Fig. 5). No Roth spot was observed in the fundus. The diagnosis of infective endocarditis on the native mitral valve was maintained, complicated by several septic emboli. The initial treatment involved a dual-antibiotic therapy based on ceftriaxone 2 g/day and gentamicin 120 mg/day chosen based on the antibiogram and antibiotic availability in the hospital at that time. After one week of antibiotic therapy, the clinical and biological evolution was favorable, with a normal and stable temperature curve (37.1 °C), a C-reactive protein that dropped to 30 mg/L, and a negative blood culture. Given the persistence of the vegetation with a size > 10mm on echocardiography and the embolic nature, the surgical indication for replacement of the mitral valve was made.

On the eighth day of antimicrobial treatment, the patient underwent mitral valve replacement with a double-winged mechanical prosthesis, specifically the ON-X prosthesis. A direct view of the mitral valve through a left atriotomy revealed significant anatomical damage to the valve, accompanied by notable vegetations on the anterior and posterior leaflets. The procedure and the postoperative period proceeded without complications. Subsequently, the patient received gentamicin for an additional week, totaling two weeks, and ceftriaxone for a duration of six weeks after the first negative blood culture. The postoperative course was uneventful. Control echocardiography demonstrated a functioning prosthesis with good wing clearance, an average gradient of 4 mmHg, and a mitral surface area of 2.3 cm^2^, without any pathological leaks. The patient was placed on curative anticoagulation with acenocoumarol and received education on preventing endocarditis, emphasizing strict hygiene and asepsis. During the six months of outpatient follow-up, the patient remained in good health. Regarding extracardiac lesions, the treatment for splenic infarction involved only analgesics with monitoring, as long as there were no complications such as abscess formation or splenic rupture. For brain abscess and vertebral osteomyelitis, the treatment was solely medical, based on antibiotic therapy, as long as there was no evidence of mass effect or involvement. The clinical evolution was favorable, with recovery from hemiparesis, and radiological assessments showed positive progress.

## Clinical discussion

The complications of infectious endocarditis are a significant source of morbidity and mortality [2]. Despite advancements in IE management [3], the number of patients experiencing at least one complication requiring surgical intervention has remained constant over time. In fact, primarily bacteria induce irreparable valvular damage. Secondly, during the acute stage of IE, vegetation particles enter the bloodstream and cause localized vascular inflammation and vascular embolism. While the majority of pulmonary embolisms are caused by particles from the right side of the heart, the bacteria-carrying particles mostly affect individuals with left-sided IE [4]. According to studies [5], there are several characteristics that increase the likelihood of embolization in infective endocarditis, including the vegetation’s size (> 10mm), motility, location on the mitral valve as opposed to the aortic valve, and a CRP level of > 40mg/l. Aerococcus Viridans (AV), as isolated in our patient, is not the most frequently isolated germ in simple or complicated endocarditis; only a few cases have been reported [6]. It is a coccus that is microaerophilic, Gram-positive, catalase-negative, and has a propensity to form tetrads. Its growth properties are comparable to those of enterococci and streptococci. Aerococci are environmental isolates that are regularly found in dust, raw vegetables, animals and their products, as well as human skin, as well as the air of residential buildings (hospitals, schools, industries, and offices) [7]. Meningitis, vertebral osteomyelitis, endocarditis, para-aortic abscess, urinary tract infections [8], bacteremia, and septic arthritis are all brought on by AV. Although the risk factors for AV systemic infections are not fully understood, granulocytopenia, oral mucositis, prolonged hospitalization, prior antibiotic therapy, invasive procedures, and implantation of foreign bodies have all been linked to severe infections with AV [9]. In all reported cases [6], vegetations were identified on the mitral or aortic valves. As in our patient, it often had a long latency period (subacute 73%); in every case recorded, blood cultures and echocardiography were used to make the diagnosis. However, there has only ever been one documented case of splenic embolization. Currently, the imaging methods used to assess embolic endocarditis include ultrasound, MRI, CT, and PET‐CT. The brain is the most common site of embolization, followed by solid organs, including the spleen, kidney, and lung. Less common sites of embolization include peripheral arteries, coronary circulation [10], and the eyes [5]. The true incidence of embolic events is unknown, with estimates ranging from 10 to 50% of IE [11]. Cerebral embolisms are sometimes inaugural and associated with the worst prognosis, with a mortality rate of 21–81% [12]. In several European studies, ischemic cerebrovascular accident constitutes 20–60% of the neurological complications of infective endocarditis, especially in the territory of the middle cerebral artery [13]. A brain abscess is more frequently a feature of acute endocarditis than subacute endocarditis. The abscesses may be single or multiple, and their clinical presentation may be that of a space-occupying lesion, toxic encephalopathy, or meningitis [14]. Involvement of the spinal cord or peripheral nerves is exceptional. Signs and symptoms related to spine involvement can be nonspecific. Patients can simply present with low back pain, a common complaint in the elderly population with degenerative joint disease [15]. Splenic and renal embolisms and certain cerebral embolisms are frequently completely asymptomatic and discovered by systematic paraclinical examinations when looking for remote complications [16]. Appropriate antimicrobial therapy remains the favorite treatment to prevent embolic endocarditis. However, there is no evidence to suggest that prolonged antimicrobial treatment can effectively reduce the incidence of embolic endocarditis. Guidelines recommend that the selection of antibiotics be based on the sensitivity of the newly isolated bacteria, and the duration of antibacterial treatment is usually two to six weeks [5]. According to the 2015 European Society of Cardiology guidelines [17], the primary indications for the use of surgery to prevent embolic endocarditis are the presence of persistent vegetations > 10 mm and one or more previous episodes of embolic endocarditis despite appropriate antibiotic therapy (Additional files 1 and 2).

## Conclusions

Complications significantly worsen the prognosis of infective endocarditis, rendering it more serious. The presence of septic and hemodynamic consequences in multi-complicated endocarditis is associated with a particularly poor prognosis and a high fatality rate. Surgical intervention is often imperative in conjunction with timely and appropriate antibiotic therapy to ensure a greater likelihood of survival and eliminate potential life-threatening risks. Remarkably, our patient serves as an example of someone who, despite encountering multiple challenges, has shown positive progress in their recovery.

### Supplementary Information

**Additional file 1:** Video in apical section on echocardiography showing the severity of mitral regurgitation flow.

**Additional file 2:** Video in apical section on echocardiography showing the two vegetations on the anterior and posterior mitral leaflets.

## Data Availability

Data sharing is not applicable to this article as no datasets were generated or analyzed during the current study. Only figures are available.

## Abbreviations

IE: Infectious endocarditis
EROA: Effective regurgitant orifice area
PISA: Proximal isovelocity surface area
AV: Aerococcus viridans
MIC: Minimal inhibitory concentration
